# The role of interplate locking on the seismic reactivation of upper plate faults on the subduction margin of northern Chile

**DOI:** 10.1038/s41598-021-00875-6

**Published:** 2021-11-02

**Authors:** Gabriel González, Francisco Pasten-Araya, Pia Victor, Yerko González, Jordán Valenzuela, Mahesh Shrivastava

**Affiliations:** 1 Departamento de Ciencias Geológicas, National Research Center for Integrated Natural Disaster Management, Universidad Católica del Norte, Avenida Angamos 0610, Antofagasta, Chile; 2 Departamento de Geofísica, Facultad de Ciencias Físicas y Matemáticas, Universidad de Chile, Santiago, Chile; 3 Helmholtz-Zentrum Potsdam, Deutsches GeoForschungsZentrum (GFZ) Potsdam, 14473 Potsdam, Germany; 4 Programa de Doctorado en Ciencias Mención Geología, Universidad Católica del Norte, Antofagasta, Chile

**Keywords:** Seismology, Tectonics

## Abstract

Quaternary deformation in the northern Chile forearc is controlled by trench parallel shortening along reactivated Mesozoic faults. Dextral strikes-slip is expressed in NW–SE striking faults of the Atacama Fault System, and reverse displacement dominates in E–W faults. This deformation results of the convergence in a concave-seaward continental margin. On September 11th, 2020, a M_w_ 6.3 earthquake and its subsequent aftershocks took place in the coastal region of northern Chile, revealing the reactivation of the deepest segment of a WNW–ESE striking upper plate fault. The reactivation of this fault occurred after the M_w_ 8.1 Iquique earthquake, and it seems to be connected to a N–S interplate locking segmentation of the plate margin, which is clearly shown by the locking pattern before the Iquique earthquake. This poses the question of how heterogeneous locking influences upper plate seismicity and how it relates to trench-parallel shortening.

## Introduction

Geodetic observations have shown with unprecedented details how plates are locked at subduction zones^1–3^. These observations have revealed that Interplate locking is fundamental for earthquake nucleation and earthquake propagation^4–6^. However, an unexplored aspect is how interplate locking can influence the earthquake generation in the upper plate. As, interplate locking is a transient property that is strongly dependent on the subduction earthquake cycle, the interlink between locking and the permanent deformation of the upper plate has remained unexplored. Sippl et al.^7^ presented a high-resolution relocation of upper plate earthquakes in northern Chile, detecting a clustered background seismicity in areas of low-interplate locking.

**Figure 1:**
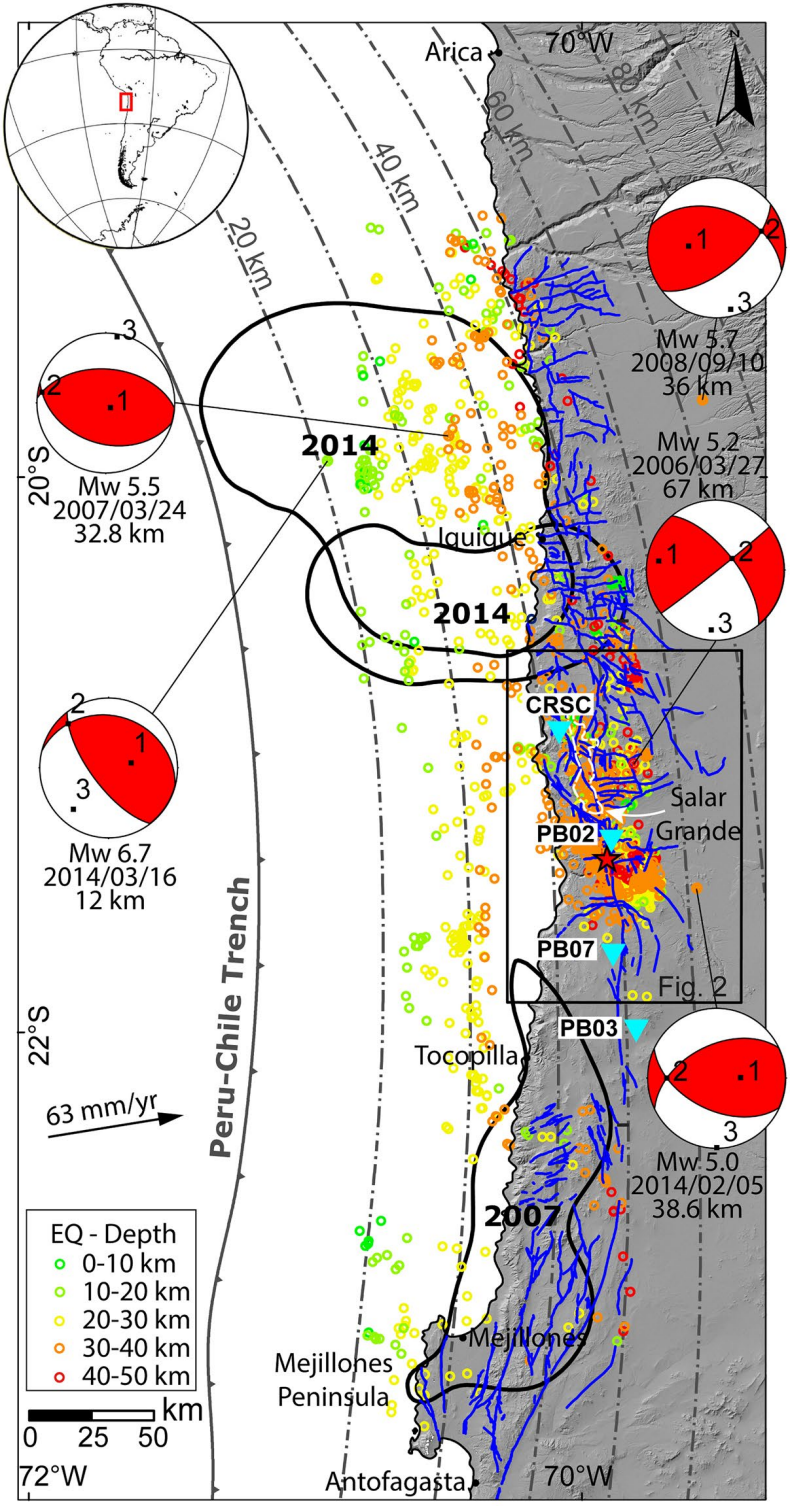
Location of the study area (inset upper corner left side) and upper plate faults in northern Chile; the epicentre of the main M_w_ 6.3 earthquake is indicated by a red star, relocation using SEISAN, (https://www.geo.uib.no/seismo/SOFTWARE/SEISAN/). Focal mechanisms provided for the CMT catalogue of M_w_ > 5.5 upper plate earthquakes that occurred from 1995 to the present. The numbers represent 1 = tensional axes, 2 = intermedia axes, and 3 = compressional axis calculated with the software FaultKin (http://www.geo.cornell.edu/geology/faculty/RWA/programs/faultkin.html). The black contours represent > 0.5 m coseismic slip for the M_w_ 8.2 Iquique earthquake (2014) and the M_w_ 7.7 major aftershock of Iquique^40^, and M_w_ 7.7 Tocopilla earthquake. Dashed grey lines show the depth of the interplate contact from Slab 2.0^41^. Circles with a colour scale show the hypocentre location of the upper plate earthquakes^7^, aftershocks of the M_w_ 8.1 Iquique earthquake were filtered. Convergence velocity calculated according to Chlieh et al.^42^. Hillshade map of northern Chile (grey background map) was generated in ArcGIS version 10.8.2 (https://www.esri.cl/es-cl/inicio) based on the Global SRTM (Shuttle Radar Topography Mission of NASA) 1 Arc-Second DEM (https://earthexplorer.usgs.gov/).

In northern Chile, several trench-orthogonal reverse faults and trench-oblique strike-slip faults of the Atacama Fault System are heavily expressed in the topography^8,9^, suggesting the contribution of the trench-parallel shortening to the Quaternary upper plate deformation (Fig. 1). Despite the recorded background seismicity, there is no clear understanding if these upper plate faults are able to generate damaging earthquakes. Since 2005 to the present, five M_w_ > 5.0 earthquakes with focal mechanism characterized by N-S trending compression and located above the interplate contact have been recorded teleseismically (https://www.globalcmt.org^10^). The depth location accuracy has been quantified for only the M_w_ 6.7 off-Iquique 2014 earthquake^11^. The rest of the other earthquakes were located sufficiently far from the interplate contact by at least 8 km (Fig. 1); therefore, it is reasonable to assume that these earthquakes are located in the upper plate. Despite the occurrence of these upper plate earthquakes, there is no evident link between these seismic events and a particular upper plate fault. On September 11, 2020, a M_w_ 6.3 earthquake struck the coastal area of northern Chile, between Iquique and the Loa River (Fig. 1). Because this earthquake was the second largest in magnitude upper plate earthquake occurring in the last 15 years, we carried out an intensive seismological study to characterize the seismic source of this earthquake. A distinctive aspect of this earthquake is the focal mechanism reported by Geofon (http://geofon.gfz-potsdam.de/eqinfo/event.php?id=gfz2020rwqv) and the USGS (https://earthquake.usgs.gov/earthquakes/eventpage/us7000blm2/executive), which is characterized by trench-transverse nodal planes. The compressional character of the reported focal mechanisms for the mainshock clearly warns of the potential reactivation of upper faults by N–S shortening. In this contribution, we relocate the main earthquake and aftershocks to illuminate the rupture zone and calculated the moment tensor solution for the mainshock and aftershocks. Through these procedures, the determination of the stress condition present during the rupture and the aftershock phase was completed. The occurrence of this earthquake raises two fundamental questions about the ability of some trench orthogonal reverse faults of the Coastal Cordillera to be reactivated by the N–S compression and whether the reactivation of these faults is in some way related to the locking structure of the plate interface. The Cascadia subduction zone in the northwest of the North American Plate and also the Hokkaido segment in the northeastern Japan subduction zone are analogous in terms of the margin curvature of the Andean Orocline margin^11^. Therefore, the result of this contribution can add to a better understanding of the active faulting process in this type of curved margin, and in turn, studying this upper plate deformation may help us understand the plate interface regime.

### Seismotectonic and structural setting

The northern Chile seismotectonic setting is dominated by the interaction of the South American Plate and the Nazca Plate along the Andean subduction zone, which influences the tectonic processes occurring along the western part of South America (Fig. 1). In particular, the coastal and offshore regions of northern Chile are located in the major seismic gap of the Andean megathrust between Mejillones and Arica^12^. This gap partially ruptured during two recent earthquakes, i.e., the March 2014 M_w_ 8.1 Pisagua earthquake^13,14^ and the November 2007 M_w_ 7.7 Tocopilla earthquake^15^. Both earthquakes released nearly 20% of the slip deficit that had accumulated since the last large earthquake that occurred in northern Chile in 1877^14^. Recently, Sippl et al.^7^ presented a summary of the seismicity of northern Chile between 2007 and 2014, illuminating in great detail the seismogenic structures present in northern Chile. According to this work, upper plate seismicity is widely distributed beneath the Coastal Cordillera near the Salar Grande, where the northern termination of the Atacama Fault System is located (Fig. 1). The described upper plate seismicity ends sharply south of Loa River, revealing NS segmentation of the upper plate seismicity in the Coastal Cordillera.

**Figure 2:**
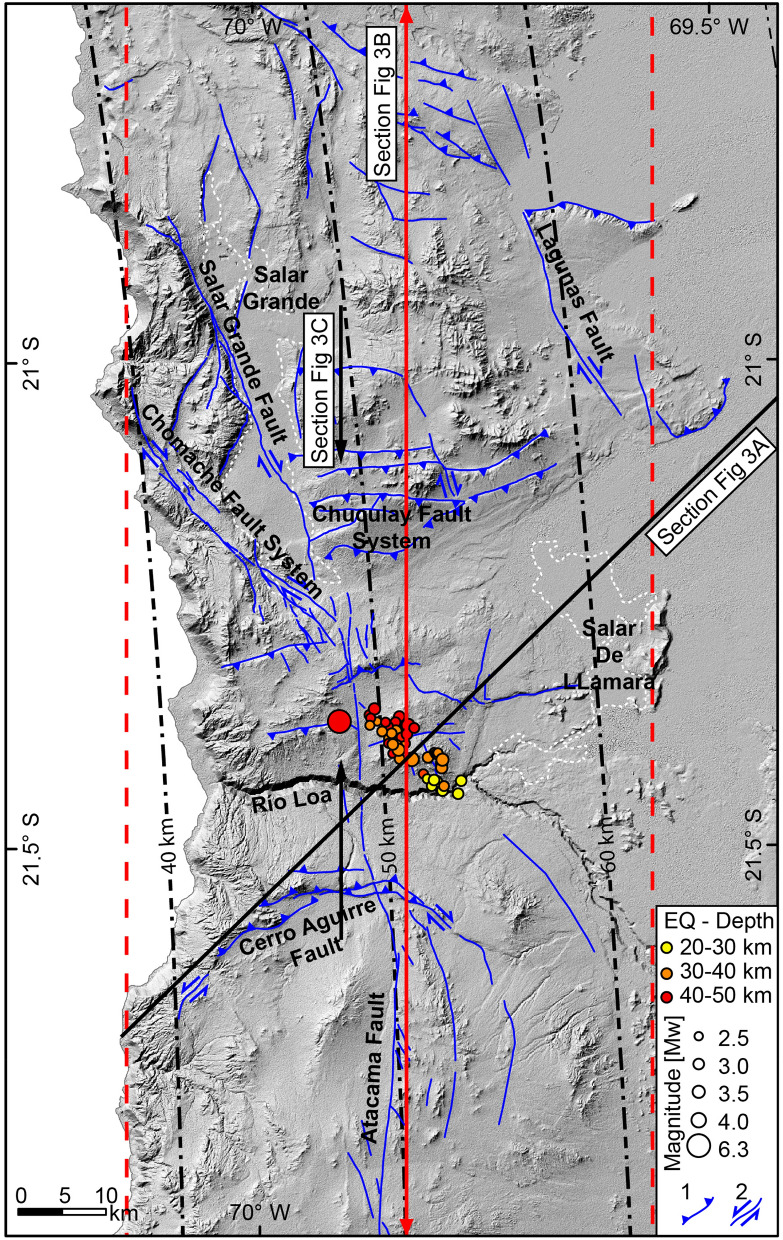
Main upper plate faults of the Coastal Cordillera of northern Chile. Grey background is a hillshade image generated by ArcGIS 10.8.2 (https://www.esri.cl/es-cl/inicio) based on the 30-m DEM provided by open-access ALOS PALSAR data (https://search.asf.alaska.edu/#/). The circles represent the HypoDD-relocated hypocentre of the aftershock sequence of the M_w_ 6.3, the diameter is scaled to the moment magnitude, and the colour scale represents the focal depths. The black lines with arrows represent the profile in Fig. 3C, and dashed vertical red lines represent the border of the swath profile containing the hypocentre locations of Sippl et al.^7^.

**Figure 3:**
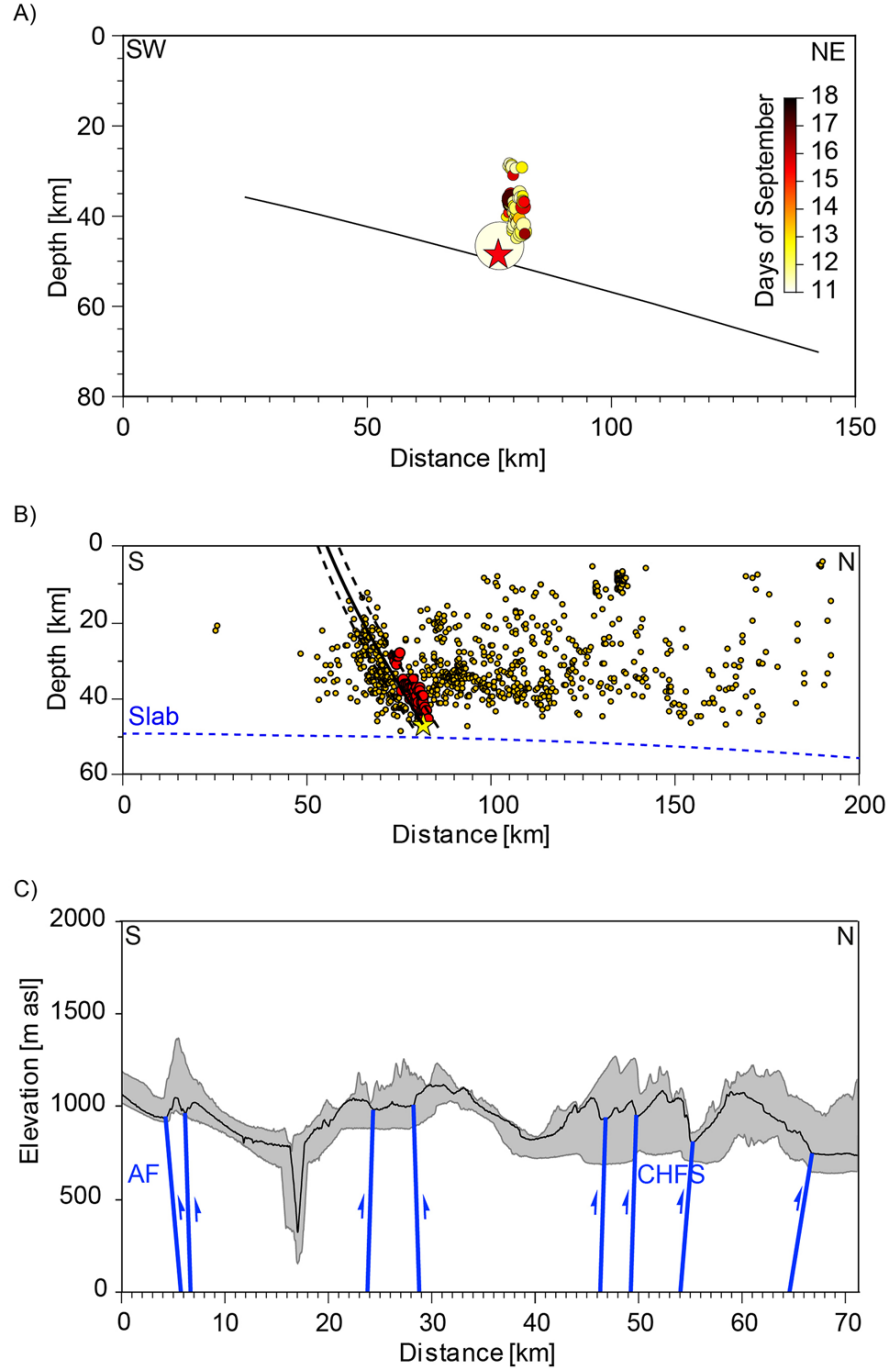
**(A)** Vertical section in the NE-SW direction showing the distribution of mainshocks and aftershocks. The colour scale represents the number of days after the mainshock. The thin black line represents the interplate contact projected in the section. **(B)** The NS vertical section shows the distribution of upper plate earthquakes (orange circle) of the catalogue of Sippl et al.^7^ and red circles show the HypoDD relocated aftershocks of the M_w_ 6.3 event (yellow star). The segmented blue line projected in the section is the slab 2.0 model. The black continuous and segmented lines represent the Cerro Aguirre Fault in the section. **(C)** The NS swath profile of the topography of the Coastal Cordillera shows the interpretation of trench-orthogonal reverse faults. The swath profile was created with ArcGIS 10.8.2 (https://www.esri.cl/es-cl/inicio) based on the 12.5-m DEM provided by open-access ALOS PALSAR data (https://search.asf.alaska.edu/#/).

The Atacama Fault System is the major structure of the Coastal Cordillera of northern Chile, which extends regionally for 1000 km between La Serena and Iquique (25°S–20°S). This fault system was formed during the Early Cretaceous as a response to oblique convergence between the Phoenix Plate and the South American Plate^16^. Oblique convergence occurring during the Early Cretaceous was able to generate trench parallel arcuate faults^17^. Later, during the Quaternary, reactivation of the Atacama Fault System was dominated by normal faulting in the Coastal Cordillera near Antofagasta and strike-slip faulting between Loa River and the Salar Grande^18^. In the latter area, the main branches of the AFS strike NW–SE and coexist with several kilometre-scale E-W-oriented reverse faults, which at the eastern part of the Salar Grande form the Chuculay Fault System^8,19^. These structures are well exposed between 21.5°S and 19.3°S, controlling the faulted topography of the Coastal Cordillera (Figs. 2 and 3c). According to previous studies^8,9^, these faults formed during the Mesozoic as extensional faults, which were reactivated as reverse faults during the present-day tectonic regime^9^. Kinematic analysis and faulting age determination^20^ demonstrated that the NW–SE striking faults, including the Chomache Fault and the Salar Grande Fault, were reactivated by dextral displacements after the Pliocene (Fig. 2). In the coastal region between Iquique and Arica, two E-W trending reverse faults cut Pleistocene marine terraces^9^, attesting to the Quaternary reactivation of these trench-orthogonal faults. A local seismic network composed of 21 short-period stations installed between 2010 and 2012 in the Salar Grande area recorded 31 upper plate earthquakes of M_w_ 0.6–2.7^11^. These earthquakes have reverse and strike-slip fault mechanisms with nodal planes striking transversely and obliquely to the trench direction. The P axis is mainly NS orientated. This seismicity extends from 7 km to a depth of 50 km, indicating that the occurrence of an upper plate stress field is dominated by N–S compression.

### GPS observation

Northern Chile is densely covered by GPS stations of the Integrated Plate Boundary Observatory (IPOC), which is dedicated to the study of earthquakes and deformation of the active continental margin of Chile^21^. GPS observations are based on survey mode (sGPS) and operated continuously (cGPS). Interseismic velocity is characterized by an ENE displacement of GPS sites parallel to the convergence velocity over the entire forearc area, being higher near the coastline and decreasing eastward (Fig. S1). This interseismic pattern was transiently affected by the two large subduction earthquakes that occurred in northern Chile, the 2007 M_w_ 7.8 Tocopilla earthquake and the 2014 M_w_ 8.1 Iquique earthquake. Presently, the entire section of northern Chile is relocked, and a clear interseismic stage is well defined^22^.

The image of interplate locking prior to the Iquique earthquake in northern Chile has been reproduced by several research groups (Fig. S1). In particular, Metois et al.^23^ defined an along-strike segmentation of locking, which is characterized by a northern segment (Camarones Segment 19°S-20.16°S) separated from a southern segment (Loa Segment 20.4°S–22.4°S) by a low coupled zone near Iquique (20.15°S Iquique LCZ). A similar interplate-locking structure was resolved by the interseismic model of Hoffmann et al.^22^. In this model, the Iquique LCZ is located at 20.92°S–21.36°S, displaced southward with respect to the definition of the model of Metois et al.^23^. The epicentre of the 2020 M_w_ 6.3 Loa River earthquake was located above this low locked zone. Since the locking model of Hoffmann et al. uses three components (E, N and Up) of interseismic velocities, the resolution of this model is more appropriate for the analysis of the interseismic velocity variation that we include in the discussion section.

## Methods

The area in which the M_w_ 6.3 Loa River Earthquake occurred is well covered by seismic stations (Supplementary Fig. S2) of the multiparameter IPOC observatory (https://doi.org/10.14470/PK615318). We built a seismic catalogue including the principal event and 9 days of aftershock sequences, detecting a total of 83 aftershocks (Supplementary Tables TS1). The arrival times of the P- and S-waves for each event were obtained manually using the SEISAN v12.0 linux 64.tar.gz (^24^, https://www.geo.uib.no/seismo/SOFTWARE/SEISAN/). When the arrival times were obtained, we relocated the events using the double-difference programme (HypoDD^25^) and the 1D velocity model of Husen et al.^26^. The final catalogue consists of 50 events (Supplementary Table TS2), with an average horizontal error of 389.40 m and 254.16 m (N–S and E–W, respectively) and an average vertical error of 339.91 m considering a root-mean-square (RMS) < 0.3. Because we use HypoDD, the errors are relative instead of absolute errors. This is important to consider because the location of the aftershock cluster depends on the location of the main event. All the principal agencies that have reported the location of the main event coincide with the position of the main event obtained in our work. Therefore, we emphasize that the relocation of the aftershocks is substantial enough in resolution for our analysis. The moment magnitudes of the catalogue, which vary in the range of 2.8 < M_W_ < 6.3, were computed for each event by using SEISAN’s SPEC spectral analysis software^24^.

We determined the focal mechanisms of the 84 events using the polarities of the P-wave (i.e., dilation or compression) and the SV/P, SH/P, and SH/SV amplitude ratios. The polarities were directly obtained from the vertical components of the seismograms for each station. As the polarities could be clearly detected, the use of a filter was unnecessary. The amplitude ratios were obtained from both the vertical and horizontal components filtered between 1 and 5 or 5–10 Hz. Based on the polarities and amplitude ratios, we computed the focal mechanisms for events with more than seven polarities using the FOCMEC programme^27^. The take-off angles were calculated with the velocity model^23^. From the catalogue including the 84 events with a focal mechanism, we select those earthquakes relocated by using HypoDD. As a result, we obtained a total of 49 relocated aftershocks and their respective focal mechanisms (Table TS2). The focal mechanisms^28^, including nodal planes, slip vector and kinematic axes, were denoted by FaultKin software v.8.1.2 (http://www.geo.cornell.edu/geology/faculty/RWA/programs/faultkin.html).

The epicentral area of the M_w_ 6.3 earthquake is covered by four cGPS sites, CSRC, PB02, PB07 and PB03 (Fig. 1), providing key information on crustal deformation. In particular, two cGPS sites (PB02 and PB07) form an excellent baseline to reproduce the strain rate in the area overlying the hypocentre of the M_w_ 6.3 earthquake. We processed 2008–2018 daily time series of these cGPS sites to characterize the interseismic velocity with GAMIT/GLOBK postprocessing software^29,30^. None of the GPS sites had a complete record of localization for this period; in some cases, the record started later, or there were missing data during the period (see Fig. S3). cGPS data later than 2018 are not available for all of these stations; therefore, our analysis does not cover the time when the M_w_ 6.3 earthquake occurred. Since the northern Chile region during this period was affected by the 2014 M_w_ 8.1 Iquique earthquake, we divided our analysis into two datasets. One dataset includes the period between February 28, 2008, and January 1, 2014. The other dataset includes the period between April 1, 2015, and January 1, 2018. With this strategy, we discarded the dataset affected by the preseismic stage and the afterslip of this earthquake. In the processing of the GPS data, we used the best possible two phases to minimize the error range. In the first phase, we used 24-h sessions of day-to-day GPS site positions, picking the ionosphere-free combination and fixing the ambiguities to integer values. We used precise orbits from the International GNSS Service (IGS) for Geodynamics^31^ and IGS Tables to describe the phase centres of the antennas. We estimated one tropospheric vertical delay parameter per station every 3 h. In the second phase, we produced daily time series by constraining regional stations unaffected by the earthquake to their well-recognized coordinates in the ITRF2014, such as KOUR in French Guyana; BRAZ, BRFT and CHPI in Brazil; RIO2 in Patagonia; and GLPS on the Nazca plate^32^. We utilized the linear trajectory method^33,34^ to model x(t), and the GPS daily position time series in the east, north, and up components are:

$$\mathrm{x}\left(\mathrm{t}\right)=\sum\limits_{\mathrm{i}=1}^{{\mathrm{n}}_{\mathrm{p}}+1}{\mathrm{A}}_{\mathrm{i}}{(\mathrm{t}-{\mathrm{t}}_{\mathrm{R}})}^{\mathrm{i}-1}+ \sum\limits_{\mathrm{j}=1}^{\mathrm{nj}}{\mathrm{B}}_{\mathrm{j}}\mathrm{H}\left(\mathrm{t}-{\mathrm{t}}_{\mathrm{j}}\right)+\sum\limits_{\mathrm{k}=1}^{2}[\mathrm{Csin}(\frac{2\uppi }{{\uptau }_{\mathrm{k}}}\mathrm{ t})+\mathrm{Dcos}(\frac{2\uppi }{{\uptau }_{\mathrm{k}}}\mathrm{ t})]+\mathrm{E\,\, log}(1+\Delta \mathrm{t}-{\mathrm{t}}_{\mathrm{eq}}/\mathrm{T})$$

where A_i_ is the coefficient of polynomial functions of n_p_ maximum power, t_R_ is a reference time defined as t_0_, B is the coefficient of H heaviside jumps to simulate earthquakes and nontectonic effects, C and D are the coefficients of a truncated Fourier series to account for seasonal variations mostly induced by the hydrological cycle (t = 1 year for annual and t = 0.5 year for semiannual periods), E is the coefficient of the transient postseismic logarithmic component, t_eq_ is the time of the Pisagua earthquake (April 01, 2014), and T is a constant determining the timescale of the logarithmic transient.

## Results

The magnitude of the mainshock was recalculated as M_w_ 6.3; the main international agencies reported a moment magnitude (M_w_) of 6.2 for this event. The M_w_ 6.3 earthquake was localized at a depth of 47 km immediately above the downdip limit of the seismogenic zone (Fig. 2). The relocated aftershocks with a magnitude between 2.8 < M_w_ < 3.7 illuminated a 15 km long by 20 km wide reactivated area extending 28 km to 46 km in depth. The location of this reactivated area clearly shows that the main rupture is located in the upper plate (Figs. 2, 3a,b). The aftershocks conform to a plane, which is very well defined in a cross-section in a map view; they strike 315° and dip 75° to the northeast (Figs. 2 and 3a). The temporal variation in the aftershocks shows that the seismicity during the first days propagated updip and downdip along this plane; subsequently, the aftershock activity was concentrated in the central part of this plane (Fig. 3a). When we plotted the aftershock sequence along the rupture plane, we defined a 24.2 km long and 11.4 km wide ellipse plunging 45° to the northwest. We estimated a rupture area of 217 km^2^ for this reactivated plane. According to the regression of magnitude and area^35^, we calculated that the mainshock slip covered an area of 500 km^2^. We used the relation Mo = μAd to estimate a 0.8 m average coseismic slip for the mainshock.

**Figure 4:**
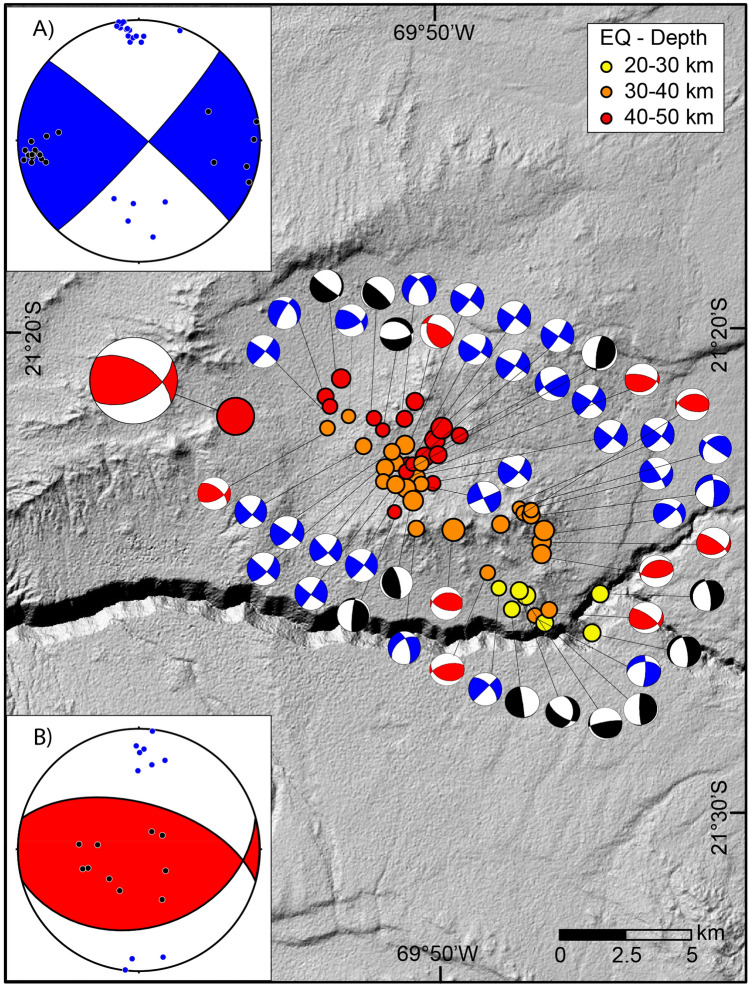
Summary of focal mechanisms of the M_w_ 6.3 main event and aftershocks based on FOCMEC programme^27^. **(A)** Composite moment tensor solution for strike-slip aftershocks showing the compressional axes (blue circles) and tensional axes (black circles), **(B)** composite moment tensor solution for reverse aftershocks. Composite moment tensor solutions are based on FaultKin software (http://www.geo.cornell.edu/geology/faculty/RWA/programs/faultkin.html). Black featured moment tensor solutions represent tensional aftershocks. The background hillshade map was generated in ArcGIS 10.8.2 (https://www.esri.cl/es-cl/inicio) based on the 12.5-m DEM provided by open-access ALOS PALSAR data (https://search.asf.alaska.edu/#/).

The obtained focal mechanism of the M_w_ 6.3 mainshock features two nodal planes oriented in a highly oblique manner compared to the megathrust strike (Figs. 1 and 4). Nodal plane 1 is a reverse-left-lateral fault; it has an azimuth of 062° and dips 54° to the southeast. The slip vector has a rake of 135°. Nodal plane 2 (NP2) is a reverse-right-lateral fault striking 302° and dipping 56° to the northeast. The rake of the slip vector is 45°. Based on the orientation of the aftershocks, we conclude that nodal plane 2 represents the active fault during this earthquake. The resulting P-axes of this focal mechanism have a trend of 2° and a plunge of 1°, whereas the T-axes have a trend of 271° and a plunge of 55°. The orientation of these two axes indicates that the mainshock kinematic regime is dominated by N–S compression.

The composite fault mechanism of the 83 aftershocks shows that the stress condition is dominated by strike-slip deformation with a nearly N–S-trending compression, the P axis is N–S trending and plunges 7°, and the T axis trends 267° with 9° of plunging (Supplementary Fig. S4). A detailed inspection of the focal mechanism of the relocated events shows that these events can be grouped into two types. The first group is represented by 28 aftershocks dominated by a strike-slip regime with mean nodal planes characterized by an azimuth of 311° and dip 86° to the northeast and an azimuth of 42° and dip 85° southeast (Fig. 4). The first nodal plane is subparallel to the strike of the activated postseismic structure defined by the correlation trend of aftershocks. The average P-axis of this group trends 356° and plunges 1°, and the T-axis trends 267° and plunges 6°. The second group is integrated by 9 aftershocks with a dominant reverse fault focal mechanism. The composite fault mechanism of this group has two nodal planes: one striking 285° and dipping 58° to the north and the other striking 75° and dipping 35° to the south. The average P-axis of this composite focal mechanism trends 3° and plunges 12°, and the T-axis trends 234° and plunges 71° (Fig. 4). These two groups of aftershocks indicate that the general stress state during the aftershock sequence was dominated by an N–S-trending subhorizontal contraction, which is similar to that represented by the moment tensor solution of the mainshock. The other 12 aftershocks show extensional focal mechanisms with tensional axes parallel to the trench normal. These aftershocks strike mostly N–S and are located at the tip of the cluster of aftershock-reactivated surfaces. The focal mechanism of the aftershock consistently shows parallel trench compression as a dominant stress condition for the main event and the aftershocks.

**Figure 5:**
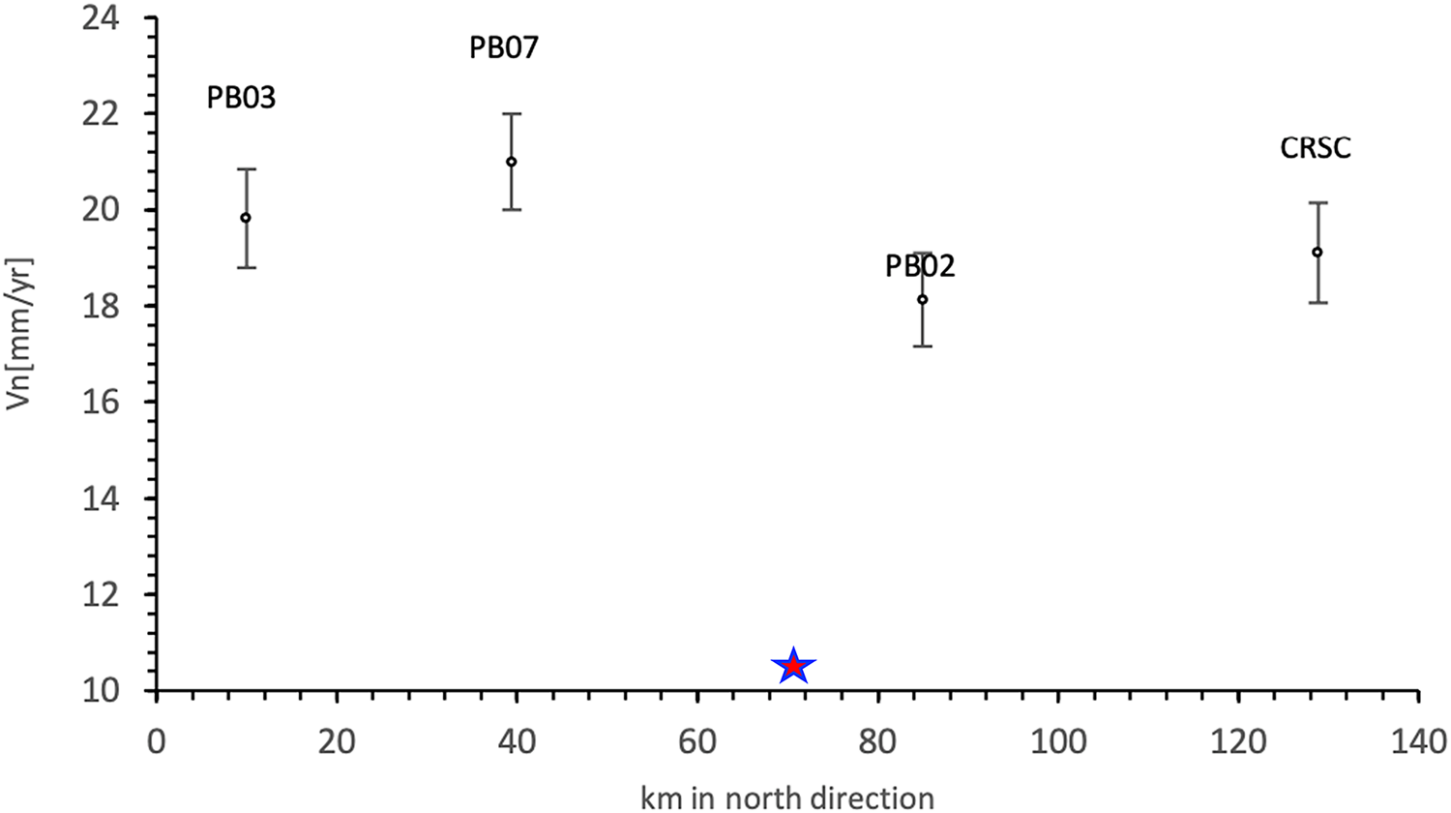
Latitudinal variation in the northern component of the interseismic velocity of CRSC, PB02, PB07 and PB03. The strongest gradient in the velocity is given by PB02 and PB07, which are located at the same distance with respect to the trench. Since PB03 and PB07 are not located at the same distance from the trench, a positive slope between both is apparent. PB03 and PB07 show higher interseismic velocities than PB02 and CRSC. Start show the location of the M_w_ 6.3 earthquake.

The cGPS data show that the interseismic velocities of CSRC, PB02, PB07 and PB03 remained with the same magnitude and identical azimuthal orientation before and after the Iquique earthquake (Table 1). The CRSC and PB02 sites exhibited slower trench parallel components of the interseismic velocity with respect to PB07 and PB03 (Fig. 5). In particular, even though PB02 and PB07 are located at the same distance with respect to the trench (138 km), PB02 moved slower than PB07. The northern components of the interseismic velocity at both stations showed a difference of 2.84 ± 1.97 mm/year before the Iquique earthquake and 2.9 ± 1.48 mm/year after this event, indicating that the section between PB02 and PB07 from 2008 to 2018 was characterized by NS contraction. We used the temporal variation in the distance between the two groups of cGPS sites to calculate the strain rate accumulated from 2011 to 2018 (Fig. 5). The baseline defined by PB02 and PB07 showed the steepest slope with a negative sign, indicating that during this period, both sites converged in the azimuthal direction of 360 with a total shortening of 34 mm during 6 years of recording. The baseline between CRSC and PB02 was subhorizontal, showing no strain accumulation, whereas the baseline defined by PB02 and PBO3 exhibited less shortening in magnitude than the baseline between PB02 and PB07. The baseline analysis indicated that the section of the forearc where the M_w_ 6.3 Loa River earthquake occurred was dominated by elastic trench-parallel shortening. This strain regime was in agreement with the N–S compression obtained by the fault mechanism of the main shock and aftershocks.

**Table 1:** Summary of the interseismic velocities of CRSC, PB02, PB07 and PB03, the last column is the integrated velocity from 2008 to 2018.

GPS sites	02/28/2008–01/01/2014 (mm/year)		01/04/2015–01/01/2018 (mm/year)		Integrated 02/28/2008–01/01/2018 (mm/year)		
	V_E_	V_N_	V_E_	V_N_	V_E_	V_N_	Vi
CSRC	26.2 ± 0.78	19.2 ± 1.01	26.4 ± 0.85	17 ± 1.06	26.3 ± 0.81	19.1 ± 1.02	32.5 ± 1.30
PB02	26.8 ± 0.66	18.2 ± 0.97	26.6 ± 0.76	18.1 ± 1.01	26.7 ± 0.71	18.1 ± 1.00	32.3 ± 1.23
PB07	31.7 ± 0.72	21.0 ± 1.00	31.7 ± 0.72	21.0 ± 1.00	31.7 ± 0.73	21.0 ± 1.01	38.0 ± 1.24
PB03	26.9 ± 0.84	19.8 ± 1.03	26.9 ± 0.85	19.8 ± 1.02	26.9 ± 0.83	19.8 ± 1.03	33.4 ± 1.32

## Discussion

According to our results, the mainshock and aftershock sequence of the M_w_ 6.3 earthquake of the 11th of September 2020, reactivated a 717 km^2^ area of a deep-seated fault structure situated in the upper plate, striking highly oblique to the trench. The focal mechanism analysis of the mainshock suggests that the rupture of the main event occurred in a fault segment striking 302° and dipping 56° to the northeast (NP2) located at the bottom of the upper plate near the plate interface. The alignment of the aftershocks in map view, as well as in a cross-section, shows the reactivation of a buried planar structure striking 315° and dipping 75° to the northeast. The aftershocks were distributed from a depth of 28 km down to near the interplate contact (Fig. 3a,b). The fault mechanism analysis indicates that a dextral strike-slip regime dominated during the aftershock propagation sequence (Fig. 4). To geometrically conciliate the slight difference (13°) between the strike direction of the main rupture and the strike of the fault plane activated by the aftershock sequence, we propose that the entire seismic sequence represents the reactivation of a 20 km long complex fault zone made of a main fault segment striking 302° in the west and 315° in the southeast. This slightly curved geometry is very common in trench-orthogonal faults delineated by the topography of the Coastal Cordillera (Figs. 1 and 2). In several locations, it is possible to identify along-strike curved faults in which the kinematics change according to the described fault orientation (Fig. 2). North of Loa River, the kinematics of the NW–SE striking upper plate faults are dominated by dextral strike-slip, whereas the most westerly striking upper plate faults are dominated by reverse faulting. Considering this context, the mainshock reactivated the most westerly portion of this curved fault zone, and the aftershocks reactivated the easternmost part.

One open question left by this earthquake sequence is whether there is some correlation between this sequence and the upper plate structure exposed at the topographic surface. Figure 2 shows that the mainshock and aftershocks localize near the N-S main branch of the Atacama Fault System and to the north of the Loa River canyon. Because the mainshock and aftershocks occurred within the north-dipping structures, a right vertical projection was not appropriated. We projected the plane delineated by the aftershock sequence (315/75NE) to the surface and the plane defined by NP2 of the main event by using the corresponding dip angle and dip direction (Supplementary Fig. S5). By this projection, we found that the surface projection of the plane given by the aftershock is close to the fault trace of the Cerro Aguirre Fault (Fig. S5), which is a large, trench orthogonal fault, that is exposed south of the Loa River canyon^8,9^. The fault trace of the Cerro Aguirre Fault is curved and composed of an E-W central segment and two bounding NW–SE and NE-SW fault segments in the eastern and western terminations of the fault trace, respectively (Fig. 2). The western segment is dominated by sinistral strike-slip movement, as shown by the offsets of several alluvial channels, whereas the eastern segment is dominated by dextral reverse displacement, as shown by the dextral separation of a paleochannel at the junction with the central segment. The aftershocks align parallel to the eastern segment of the Cerro Aguirre Fault, and NP2 is parallel to the junction zone between the central and eastern segments. The projection onto the topographic surface of NP2 is located 12 km south of the Cerro Aguirre Fault, whereas the plane defined by the aftershocks is located 4 km north of this fault (Fig. S4). Neither projection exactly matches the Cerro Aguirre Fault. Because a constant dip angle was used in our projection, small changes in the dip angle produced drastic changes in the position of the projected structure in the topographic surface. Considering this uncertainty, the most remarkable coincidence in geometry and kinematics between this seismic sequence and the upper plate faults is given by the Mesozoic Cerro Aguirre Fault. This fault is described as one of the most important trench-orthogonal faults of the Coastal Cordillera^8,9^. Since the Miocene, this fault has been reactivated and has accumulated up to 130 m of vertical displacement, forming a prominent southward facing scarp (Fig. 2). The long-term vertical tectonic uplift rate of the Cerro Aguirre Fault is 15 m/Myr from 9 Ma to the present^36^.

The Fig. 3b shows the upper plate seismicity^7^; in this projection, the aftershocks alienate within a single zone of concentrated upper plate seismicity, which coincides with the interpreted projection of the Cerro Aguirre Fault in the section. The small deviation to the north of the cluster of aftershocks is due to our relocation method, which distributes the aftershocks with respect to the mainshock. Another source of uncertainty might be posed by the velocity model used, which does not resolve the uppermost levels of the crust in great detail. The profile in Fig. 3b shows that the proposed concentration of upper plate seismicity defines a great upper plate structure, which marks the end of the upper plate seismicity southward.

**Figure 6:**
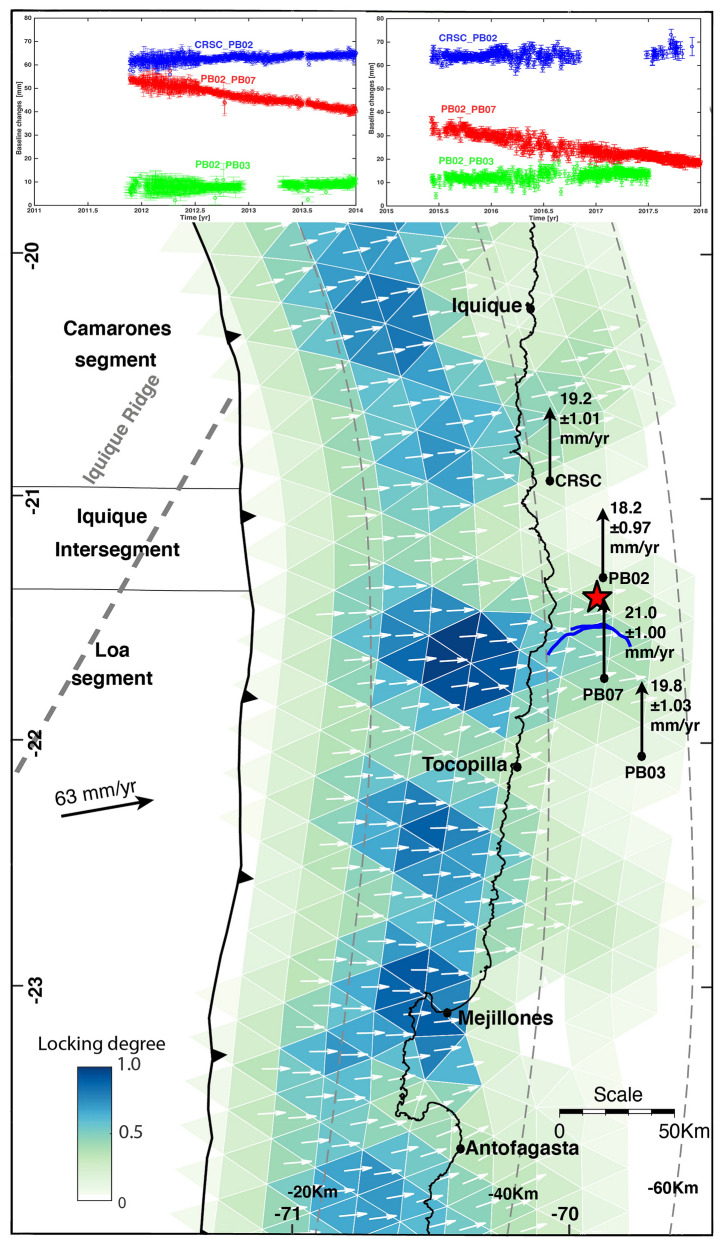
Interplate locking model before the M_w_ Iquique earthquake^22^ showing the cGPS sites used for the determination of the interseismic velocities expressed as the northern component. CRSC and PB02 are located in the intersegment Iquique LCZ and PB03 and PB07 are located in the Loa segment characterized by higher locking. The inset on the top right is the baseline formed by CRSC-PB02, PB02-PB07, and PB02-PB03.

Theoretical models explain the trench parallel shortening in concave sea subduction zones as a result of interseismic deformation along a locked curved plate margin^37^. In these models, the trench parallel component of the interseismic velocity decreases towards the maximal plate curvature, generating trench-parallel shortening. Since the plate margin where the M_w_ 6.3 earthquake occurred forms a straight section, the reduction in the trench parallel component of the interseismic velocity, i.e., trench-parallel shortening, cannot be explained by the large-scale plate curvature of the southern Peru-northern Chile subduction margin (Fig. 1). We postulated that trench-parallel shortening related to the M_w_ 6.3 Loa River earthquake was caused by a reduction in the interseismic velocity due to along-strike variation in interplate locking (Fig. 6). As we described previously, the N–S section of the Coastal Cordillera between Arica and the Mejillones Peninsula is characterized by two large segments of high locking separated by the Iquique LCZ^22^. The CRSC and PB02 sites are located in the area of low locking, whereas the PB07 and PB03 sites are situated in an area of higher interplate locking given by the Loa Segment (Fig. 6). This spatial correlation shows that the trench-parallel locking reduction from the Loa Segment to the Iquique LCZ is a key element in explaining the origin of the M_w_ 6.3 Loa River earthquake. One important aspect for validating this interpretation is whether the resolution of the locking model is sufficient to detect locking variation at the scale of distance between PB02 and PB07. This locking model is resolved with 1,016 triangular faults with an average of 170 km^2^^22^. The centroids of each patch are at a distance of 13 km from each other. Sensitive analysis based on the checkerboard test^22^ indicates that interseismic locking is well resolved south of 19° at depths between 20 and 55 km. Since PB07 and PB02 are separated by a distance of 45 km, the locking model has sufficient resolution to explain the gradient in the interseismic velocity between these two sites.

Because PB02 and PB07 form a perfect baseline oriented perpendicular to the strike of the Cerro Aguirre Fault, the gradient in the northern component of the interseismic velocity, 2.8 ± 1.9 mm/year, between these two cGPS sites is a proxy of the loading rate of the Cerro Aguirre Fault. This conceptual model suggests that trench-parallel shortening occurred in a straight section of this plate margin where high and low locking patches coexist along strike. A relevant kinematic element of this model is the obliquity of the plate convergence velocity. This ensures the existence of a trench-parallel component of the interseismic velocity of the GPS sites situated above the coupling zone. The M_w_ 6.3 earthquake demonstrated the inelastic component of the upper plate deformation during the interseismic period. This behavior was generated by the occurrence of a pre-fractured crust, where optimally oriented older structures could be reactivated by the stress generated by plate interactions.

The structure of interseismic locking in northern Chile before the Iquique earthquake has been modelled by several authors^13,22,23,38^, and all of these models reproduced the same N-S locking variation characterized by two segments of high locking separated by an intersegment of low locking referred to as the Iquique low coupled zone. This intersegment acted as a barrier for the southern rupture of the M_w_ 8.1 Iquique earthquake revealing the occurrence of mechanical anisotropy dominated by rate strengthening in this zone. At this latitude, the bathymetry of the incoming Nazca Plate is dominated by the Iquique Ridge. The occurrence of this structure is a potential explanation for this low coupled zone; in fact, a previous work^39^ explained the occurrence of the southern barrier of the Iquique earthquake as controlled by the occurrence of seamounts. Seamounts can cause coupling reduction by aseismic slip and seismic swarms. The occurrence of the Iquique Ridge, controlling the first-order frictional properties of the interplate contact, suggests that these locking structures are probably expressed long term on this plate margin and controlling, to some extent, the upper plate deformation in northern Chile.

## Conclusion

We demonstrate that the M_w_ 6.3 Loa River earthquake was generated in the deep-seated portion of an orthogonal to the trench active upper plate fault zone, which is spatially linked to the Cerro Aguirre Fault. This fault represents a key structure of the upper plate marked by a prominent fault scarp at the surface, as well as a continuously downdip fault trace marked by seismicity. During the M_w_ 6.3 earthquake and its aftershock sequences, the fault zone slipped along a plane down from mid-crustal level at 28 km depth to 47–50 km near the subduction zone interface. This fault zone forms a sharp limit of the upper plate fault seismicity of the Coastal Cordillera, separating a northern zone with widely distributed upper plate seismicity from a southern zone devoid of crustal events. This segmentation is also expressed in the faulting configuration of the Coastal Cordillera; the region exposed north of the Cerro Aguirre Fault contains a large number of oblique to the trench and trench-orthogonal faults with a Quaternary faulting record, whereas these features do not exist southward. Therefore, we propose that the Cerro Aguirre Fault represents a complex upper plate structure forming an active segment boundary of the upper plate. The observed seismic reactivation of this fault zone is controlled by N–S shortening related to trench-parallel variation of the interplate locking.

## Supplementary Information

Supplementary Figure S1.

Supplementary Figure S2.

Supplementary Figure S3.

Supplementary Figure S4.

Supplementary Figure S4.

Supplementary Legends.

Supplementary Table S1.

Supplementary Table S2.
