# Supplementary figures and images for: The role of interplate locking on the seismic reactivation of upper plate faults on the subduction margin of northern Chile
Source: Sci Rep. 2021 Nov 2;11:21444. doi: 10.1038/s41598-021-00875-6 (PMC8563723; doi:10.1038/s41598-021-00875-6)

a)

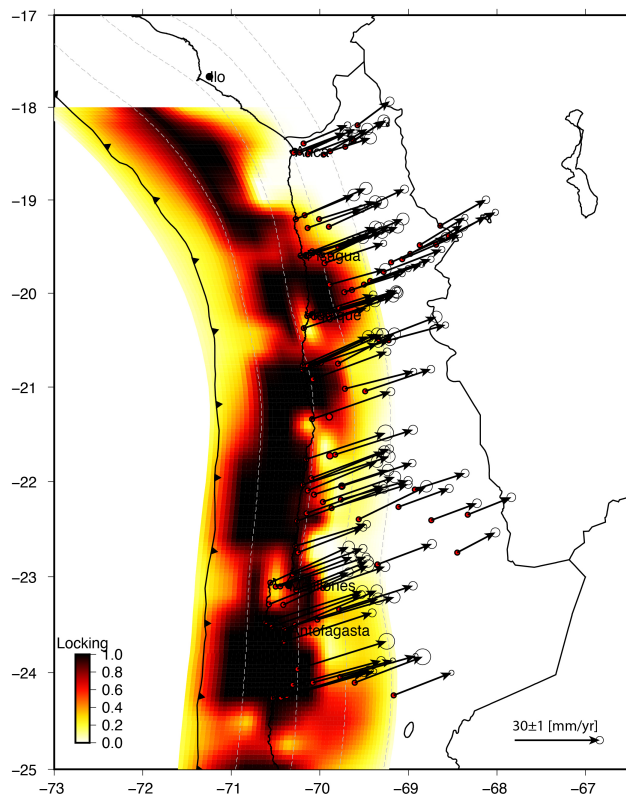

b)

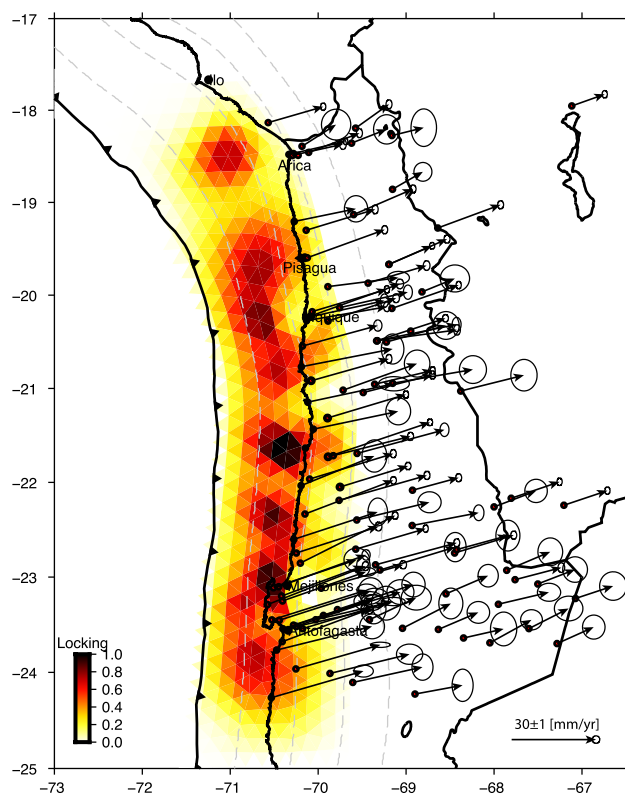

Supplement: Supplementary file 1 — Supplementary Figure S1. [file 41598_2021_875_MOESM1_ESM.pdf]

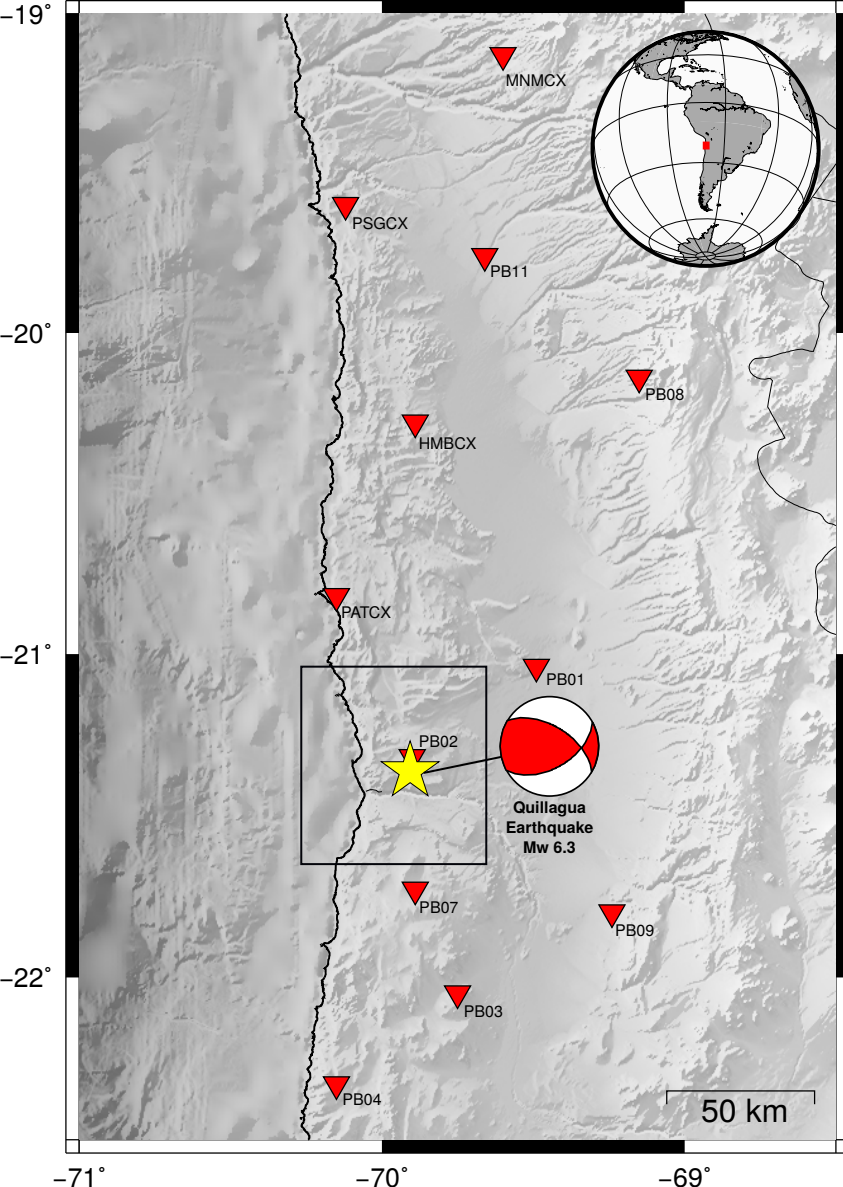

Supplement: Supplementary file 2 — Supplementary Figure S2. [file 41598_2021_875_MOESM2_ESM.pdf]

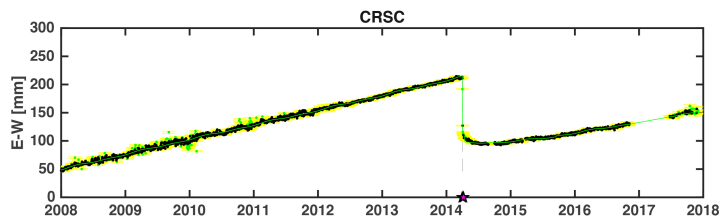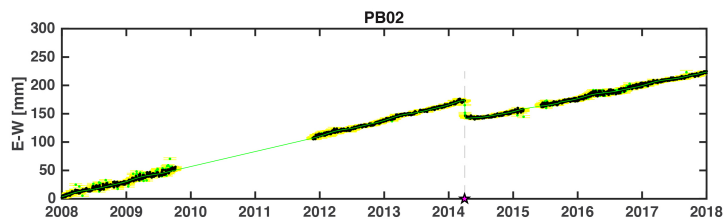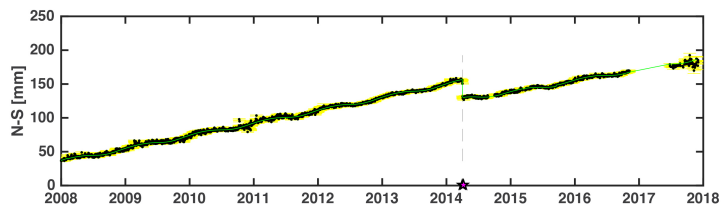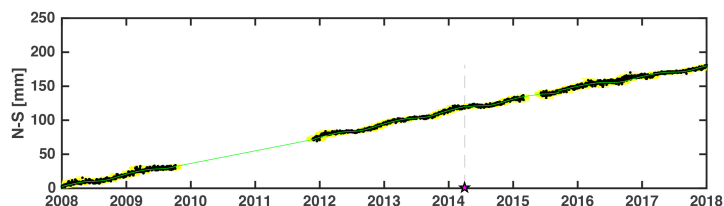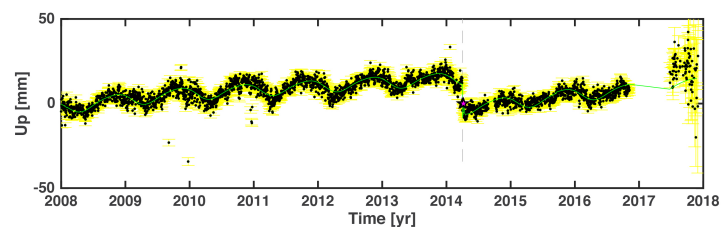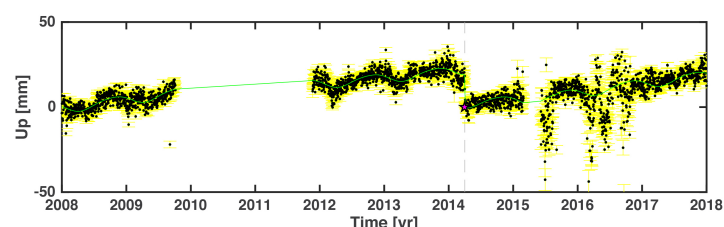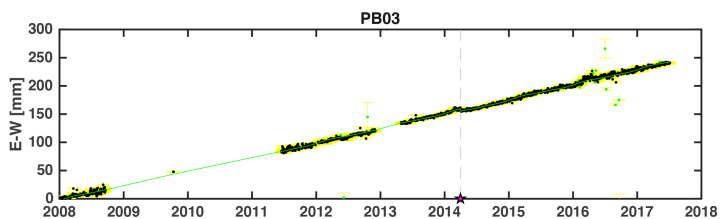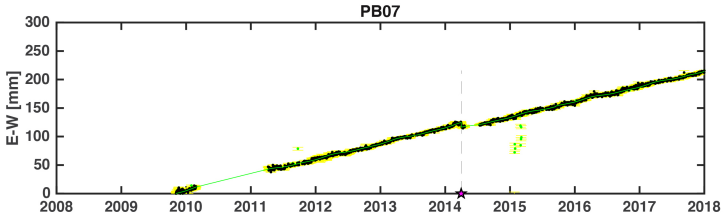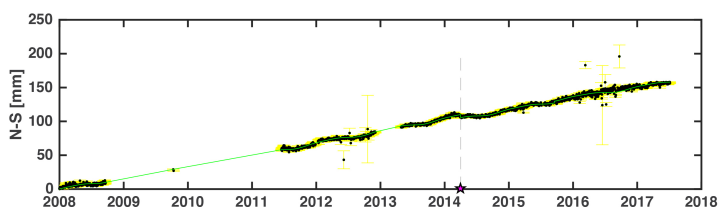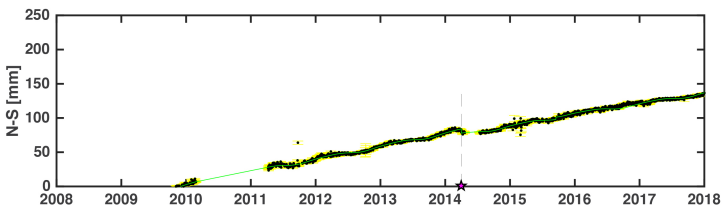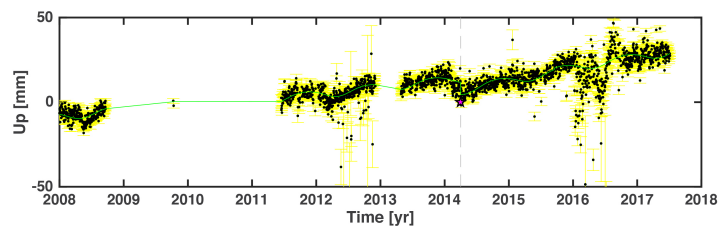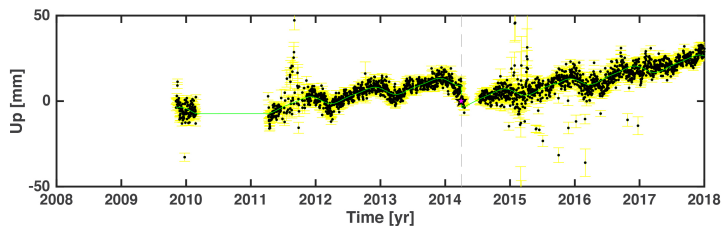

Supplement: Supplementary file 3 — Supplementary Figure S3. [file 41598_2021_875_MOESM3_ESM.pdf]

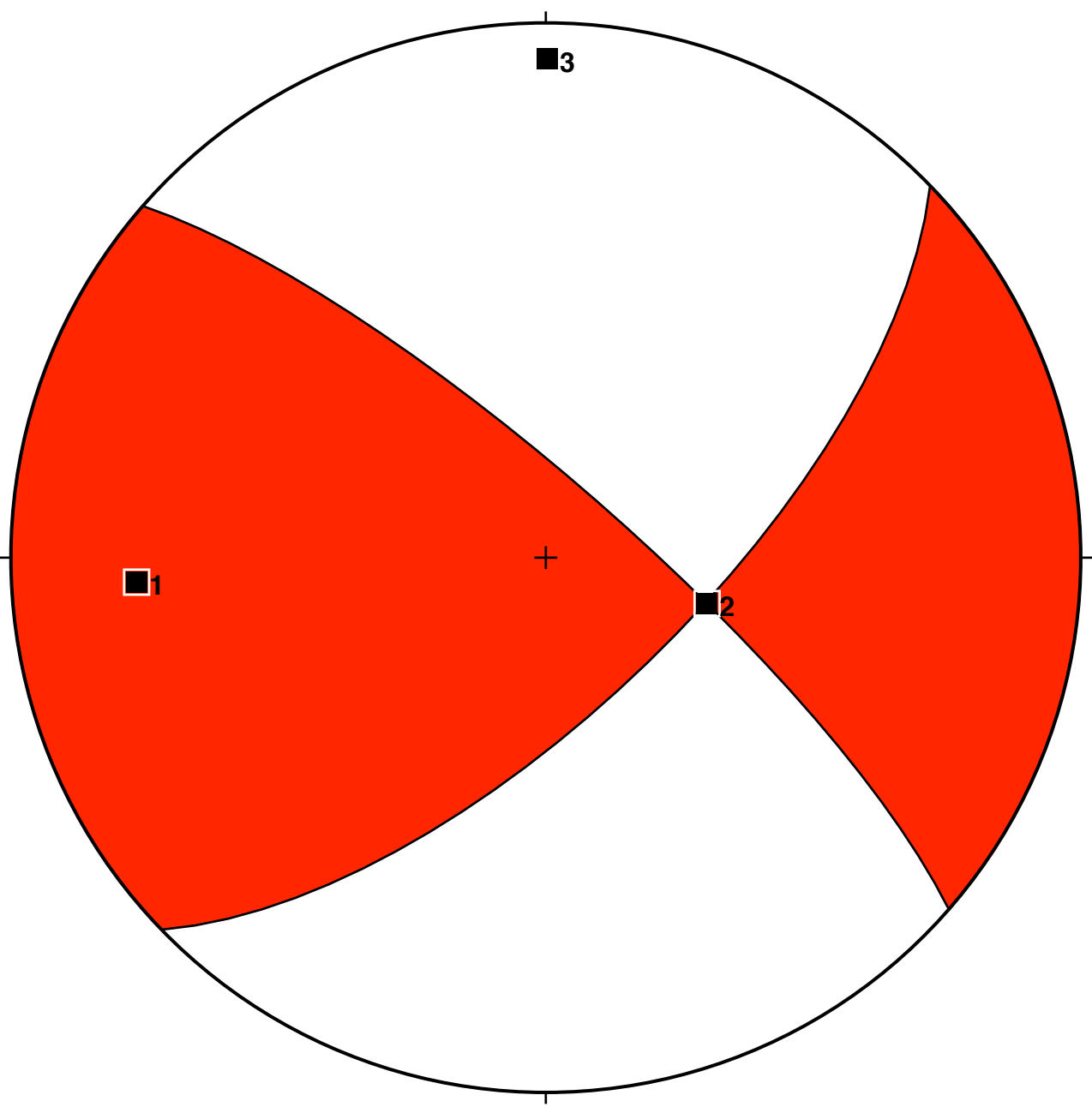

Supplement: Supplementary file 4 — Supplementary Figure S4. [file 41598_2021_875_MOESM4_ESM.pdf]

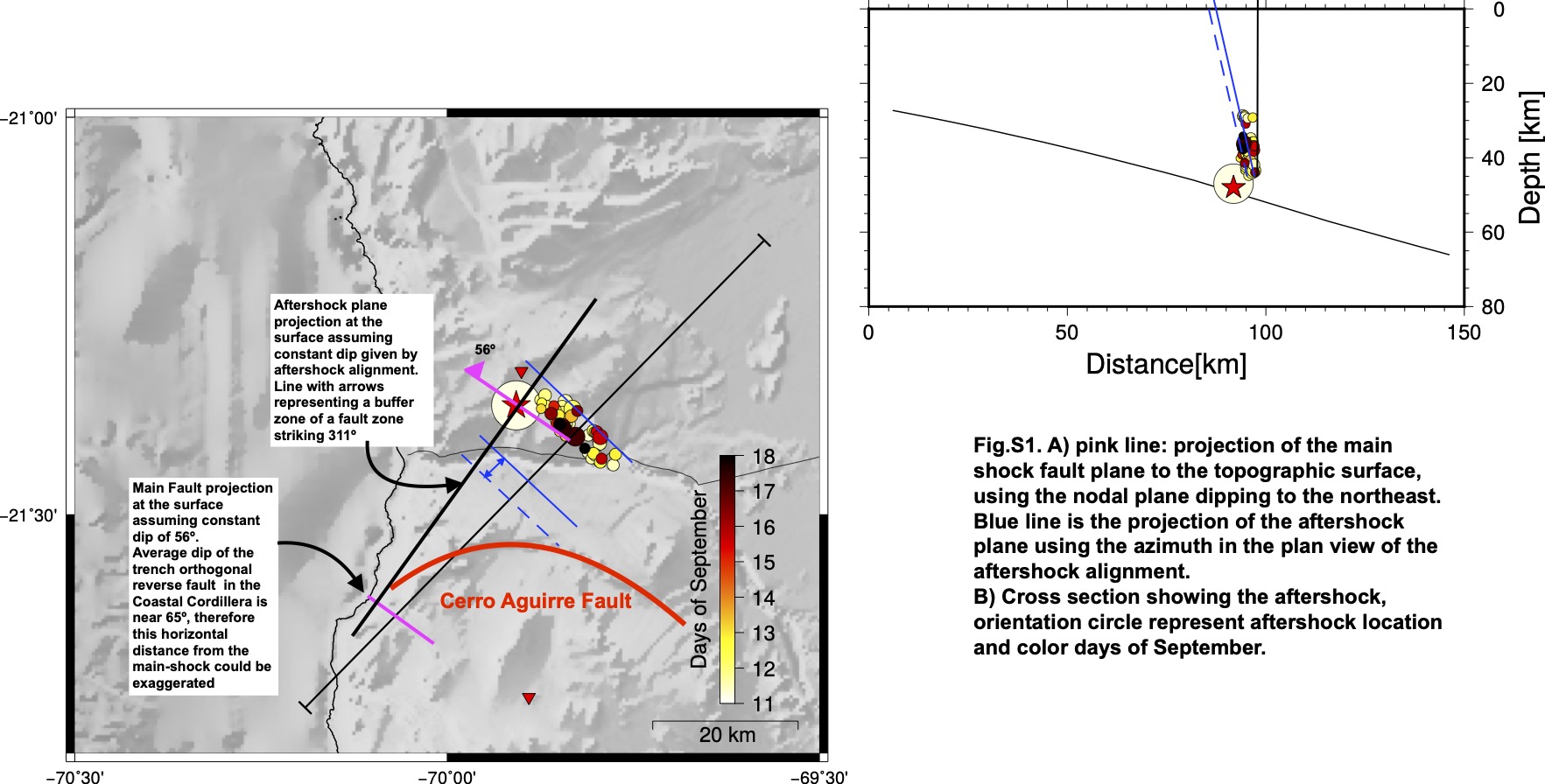

Supplement: Supplementary file 5 — Supplementary Figure S4. [file 41598_2021_875_MOESM5_ESM.jpg]
