# Supplementary material for: The role of interplate locking on the seismic reactivation of upper plate faults on the subduction margin of northern Chile
Source: Sci Rep. 2021 Nov 2;11:21444. doi: 10.1038/s41598-021-00875-6 (PMC8563723; doi:10.1038/s41598-021-00875-6)
Supplement: Supplementary file 6 — Supplementary Legends. [file 41598_2021_875_MOESM6_ESM.pdf]

Supplementary figure caption

Figure S1. a) Interseismic velocities in northern Chile plotted with the locking model of Metois et al. (24) and b) interseismic velocities in northern Chile plotted with the locking model of Hoffmann et al. (23).

Figure S2. Seismological stations of the permanent IPOC (Integrated Plate Boundary Observatory Chile) network (doi.org/10.14470/PK615318) that were close to the epicentre of the Mw 6.3 earthquake in northern Chile. We used these stations for the relocation and calculation of the focal mechanisms of the earthquake and its aftershocks shown in the main text. For both the bathymetry and topography DEM, we use the Global Bathymetry and Topography at 15 Arc Seconds (15-s resolution) file download from the website https://topex.ucsd.edu/WWW_html/srtm15_plus.html. To create the figure (plotting bathymetry, topography, and symbols) we used version 5.4.5 of the Global Mapping Tool (GMT) free program downloaded from https://www.generic-mapping-tools.org/download/. All GMT documentation can be found at http://gmt.soest.hawaii.edu/projects/gmt.

Figure S3. Time series of cGPS sites used in the manuscript.

Figure S4. Composite moment tensor solution for the 83 aftershocks. The numbers represent: 1= tensional axes, 2= intermedia axes, and 3= compressional axis calculated with the software FaultKin (26).

Figure S5. A) The pink line shows the projection of the mainshock fault plane to the topographic surface, using the nodal plane dipping to the northeast. The blue line is the projection of the aftershock plane using the azimuth in the plan view of the aftershock alignment. B) Cross-section showing the aftershock orientation. The circle represents the aftershock location and colour days of September. For a representation of the bathymetry and topography we used the Global Bathymetry and Topography at 15 Arc Seconds (15-s resolution) respectively, files downloaded from the website https://topex.ucsd.edu/WWW_html/srtm15_plus.html. To create the figure (plotting bathymetry, topography and symbols) we used the version 5.4.5 of the Global Mapping Tool (GMT) free program downloaded from https://www.generic-mapping-tools.org/download/. All GMT documentation can be found at the website http://gmt.soest.hawaii.edu/projects/gmt.

Supplementary table legends

Table S1_GGONZALEZ_RV.txt. Earthquake location using travel time including mainshock and aftershocks.

Table S2_GGONZALEZ.txt. Earthquake data, including focal mechanisms of mainshocks and aftershocks. Location data is based on HypoDD.
